# Induced Pluripotent Stem Cells (iPSCs) in Vascular Research: from Two- to Three-Dimensional Organoids

**DOI:** 10.1007/s12015-021-10149-3

**Published:** 2021-03-18

**Authors:** Anja Trillhaase, Marlon Maertens, Zouhair Aherrahrou, Jeanette Erdmann

**Affiliations:** 1 Institute for Cardiogenetics, University of Luebeck, Ratzeburger Allee 160, 23562 Luebeck, Germany; 2 DZHK (German Centre for Cardiovascular Research), Partner Site Hamburg/Kiel/Luebeck, Luebeck, Germany; 3 University Heart Centre Luebeck, 23562 Luebeck, Germany

**Keywords:** Induced pluripotent stem cells, Differentiation, Smooth muscle cells, Endothelial cells, Organoids, Vasculature

## Abstract

Stem cell technology has been around for almost 30 years and in that time has grown into an enormous field. The stem cell technique progressed from the first successful isolation of mammalian embryonic stem cells (ESCs) in the 1990s, to the production of human induced-pluripotent stem cells (iPSCs) in the early 2000s, to finally culminate in the differentiation of pluripotent cells into highly specialized cell types, such as neurons, endothelial cells (ECs), cardiomyocytes, fibroblasts, and lung and intestinal cells, in the last decades. In recent times, we have attained a new height in stem cell research whereby we can produce 3D organoids derived from stem cells that more accurately mimic the in vivo environment. This review summarizes the development of stem cell research in the context of vascular research ranging from differentiation techniques of ECs and smooth muscle cells (SMCs) to the generation of vascularized 3D organoids. Furthermore, the different techniques are critically reviewed, and future applications of current 3D models are reported.

**Graphical abstract:**

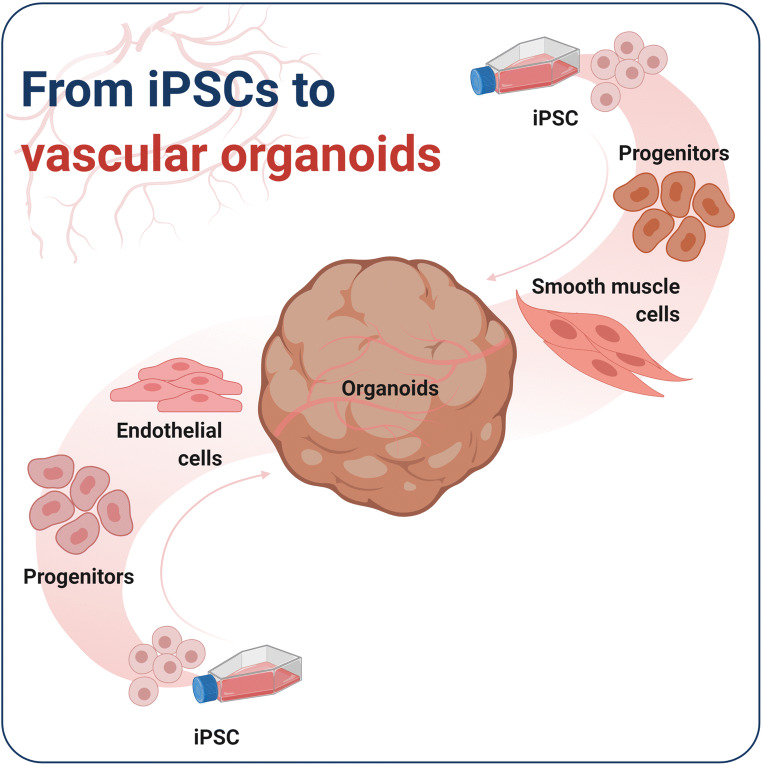

## Development and Progress of Human iPSC Technology over the Last Years

**Fig. 1:**
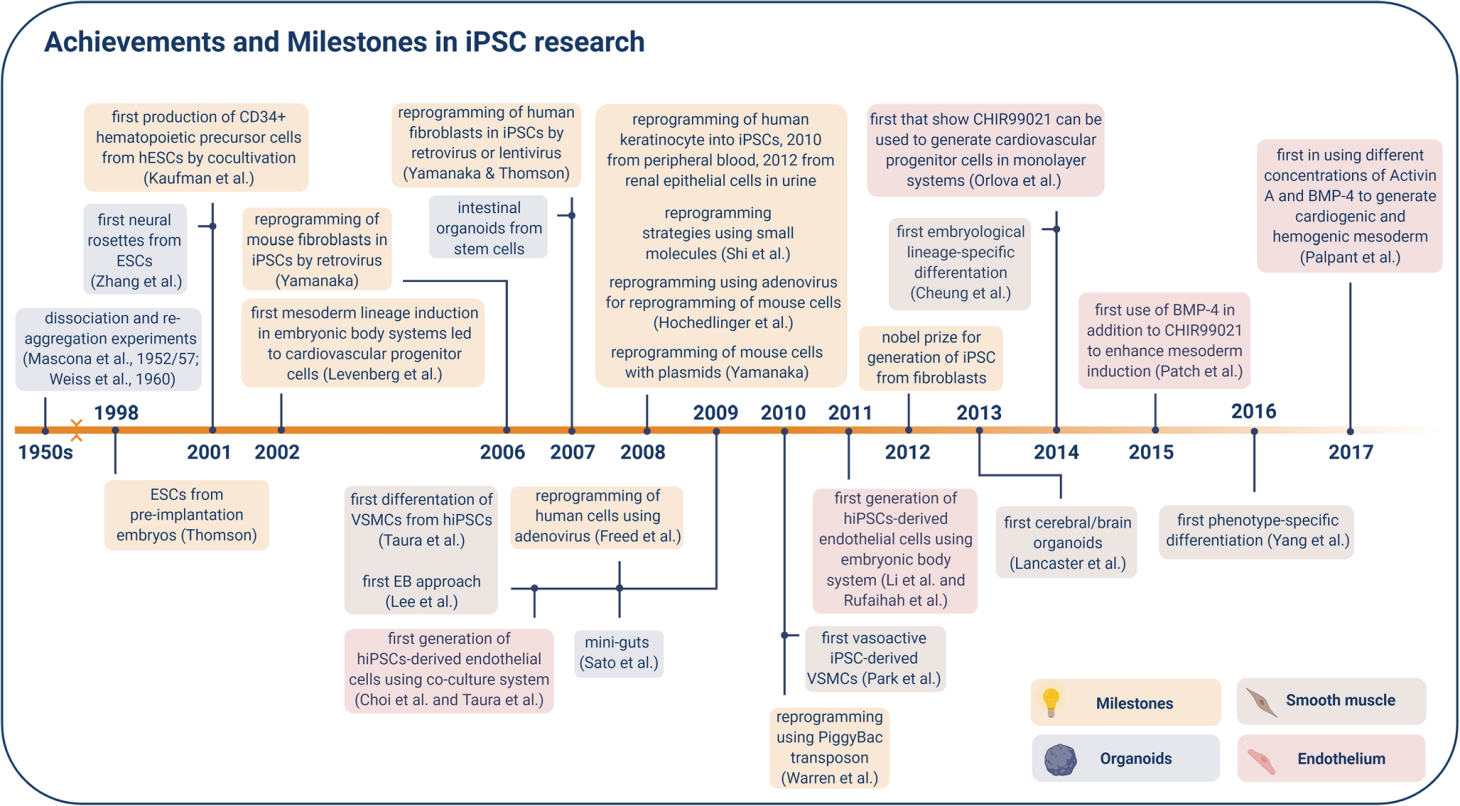
Achievements and Milestones in iPSC research. Timeline showing the achievements of iPSC technology since 1998, divided into general milestones and time-points when smooth muscle and endothelial cell derivatives were successful generated

Human induced-pluripotent stem cells (iPSCs) are a recent alternative to embryonic stem cells (ESCs) that can be generated from adult somatic tissue without the need of embryos. The technique was developed in the early 2000s when Thomson and colleagues successfully isolated human ESCs from preimplantation embryos in 1998 (Thomson, 1998). To generate iPSCs, adult somatic cells were reprogrammed by forced expression of pluripotency factors, four of which were discovered by Yamanaka and Takahashi in 2006. Takahashi and colleagues were able to reprogram mouse fibroblasts with the overexpression of Octamer-binding transcription factor 4 (*OCT4*), sex determining region Y-box 2 (*SOX2*), Kruppel-like factor 4 (*KLF4*), and v-Myc avian myelocytomatosis viral oncogene homolog (Myc, alias *c-MYC*), delivered with the help of a retrovirus [1]. In 2012, John B. Gurdon and Shinya Yamanaka were awarded the Nobel prize for Physiology or Medicine “for the discovery that mature cells can be reprogrammed to become pluripotent” (Press release. NobelPrize.org. Nobel Media AB 2020. Mon. 27 Apr 2020). Importantly, iPSCs can be generated from numerous somatic cell types, including a small skin biopsy, without putting the patient at risk of undergoing invasive surgery [2]. Nowadays, various iPSC-derived models exist to examine disease pathology and the influence of genetic risk factors on disease progression. Cell types, such as neurons, smooth muscle cells (SMCs), cardiomyocytes, and fibroblasts, as well as endothelial, lung, and intestinal cells, can be differentiated from iPSCs. However, there are only a few models for vascular cell derivatives (Fig. 1).

Previously, two-dimensional (2D) differentiation methods were the state-of-the-art technology, allowing researchers to closely monitor and manipulate single cells during maintenance and differentiation. 2D systems are efficient, simple-to-handle, and cheap [3]. However, 2D cell culture models do not reflect the natural microenvironment or physiology, including cell–cell interactions [4]. Additionally, 2D cultured cells differ in morphology and behaviour from their counterparts in vivo [4]. Therefore, three-dimensional (3D) culture systems were warranted to accurately mimic the *in vivo* environment.

Recently, 3D organoids and tissue models have been generated from iPSCs to resemble organ structure and function. Examples of complex tissues and organs modelled in 3D include Engineered Heart Tissues (EHTs) [5], Lung [6], and intestinal organoids [7], as well as so-called “mini-brains” [8]. A 3D model of the vascular system was only recently published [9].

Independent of format, one of the main questions evaluated during the emergence of differentiation methods of pluripotent stem cells (PSCs) was whether directed or undirected differentiation approaches were more feasible. For some time, it was the standard to use embryoid bodies for differentiation of specific tissues. However, this method has some disadvantages such as the lack of control over the different cell types that reside in the differentiated cell culture. It is not possible to control and regulate the differentiation path of every single cell. Cells spontaneously start differentiating, then spontaneously transmit signals to surrounding cells randomly initiating other differentiation and signalling cascades without the possibility to control the direction [10]. On the other hand, many developmental studies revealed very detailed signalling cascades leading to specific, lineage-imbedded mature cells. This method usually progresses via precursor cells of the respective tissue type and is now used in many differentiation protocols. The differentiation factors used are usually well defined and allow a cell type specific differentiation, in some cases even a differentiation of subtypes of cells [10]. In terms of organoid differentiation also two methods are known today. Organoids exclusively differentiated from PSCs often result in a heterogeneous and immature cell population. Organoids however, that were differentiated from lineage-specific precursors often mimic the physicochemical characteristics of the niche better and therefore are able to induce maturation. Further, using precursors for organoid formation makes it easier to keep control over the cells and their development within the organoid, as they are already induced into a specific lineage before formation of the organoid.

## Vessel Structure and Cell Types

**Fig. 2:**
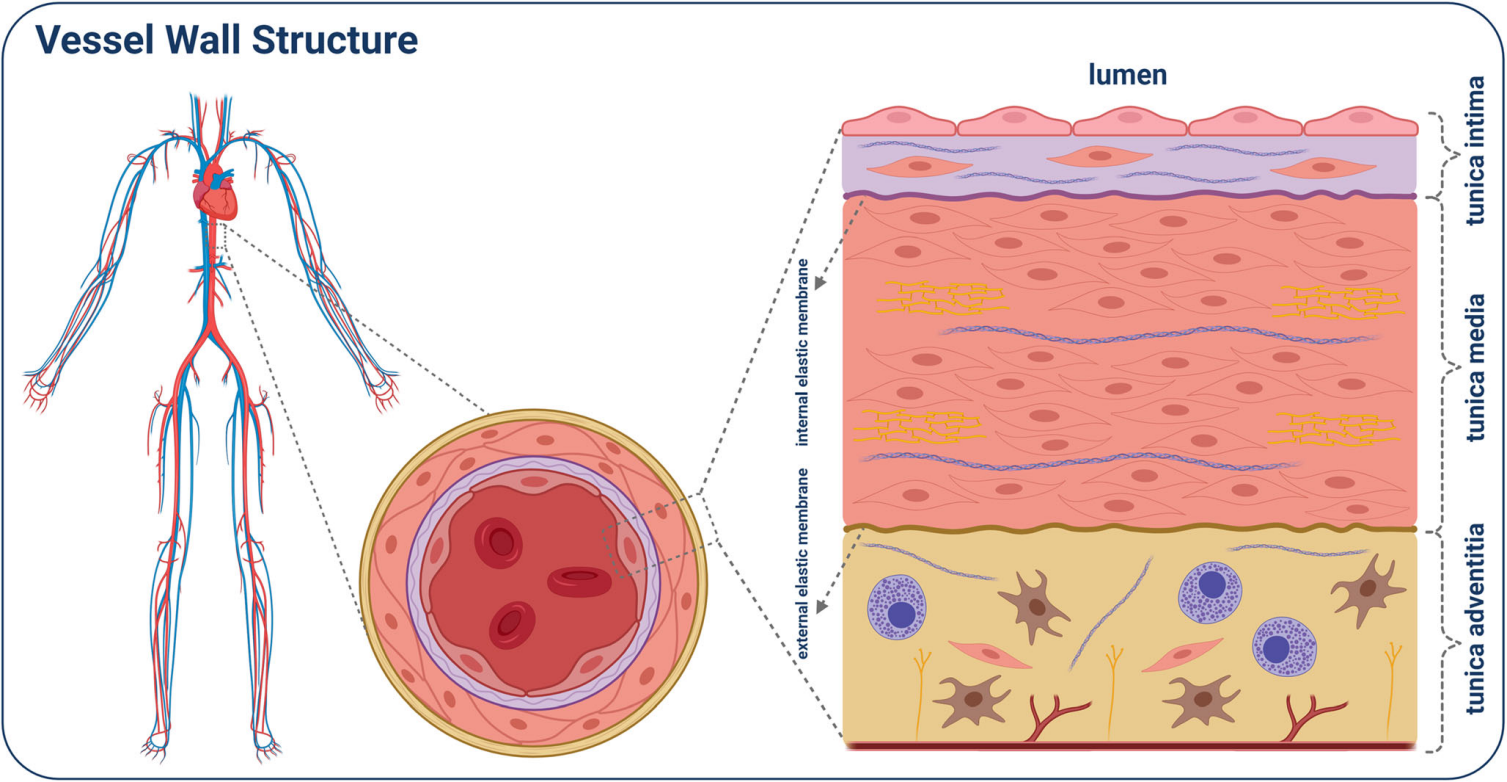
Shown is the human vascular system, with a magnified transverse section through the aorta. Further magnified is a cross-section of the vessel wall, divided into the different layers with the corresponding components

The cardiovascular system is one of our largest organs, consisting of arteries, veins, and capillaries. Cardiovascular disease (CVD) occurs primarily in arteries that transport oxygenated blood from the heart to different regions of our body. The heart artery walls consist of three different layers that surround a central lumen. Each of these layers are composed of different cells and extracellular matrix molecules. As a result, each layer may function differently in terms of homeostatic regulation and growth. The innermost layer, tunica intima, is in direct contact with the blood. It consists of a monolayer of endothelial cells (ECs), connective tissue, and resident smooth muscle cells (SMCs). The internal elastic membrane forms a barrier with the thick middle layer, the tunica media, consisting of SMCs and elastic fibres. The outermost layer is the tunica adventitia, consisting of an elastic membrane, connective tissue, fibroblasts, SMCs, mast cells, as well as *nervi* and *vasa vasorum* (Fig. 2) [11–14]. Thus, reconstruction and engineering of the vessel in vitro is challenging. Indeed, we are just beginning to understand the cell–cell interactions, especially between resident ECs and SMCs. Nowadays, SMCs and ECs can be easily differentiated from iPSCs and produced at a large scale.

## iPSC-Derived Endothelial Cells

The ECs form the innermost layer of our vessels and are responsible for the direct contact between blood and tissue. The ECs have a combined surface area of approximately 10 basketball courts [15]. Besides their role in vaso-dilatory and -constrictive processes, they are also responsible for homeostatic and signal transducing processes. Therefore, the vascular endothelium is of particular interest, especially regarding the development and progression of vascular diseases, such as myocardial infarction, stroke, neurovascular disorders, and atherosclerosis [16–18].

At the beginning of this century, scientists were reliant on the use of primary or hESC-derived ECs for vascular research. Since the discovery of iPSCs, it has been possible to produce patient-derived ECs to investigate the development and progression of cardiovascular diseases, or the individual effects of different drugs. In addition, iPSCs also offer other advantages, such as the ethical renunciation of hESCs, the avoidance of immune reactions upon implantation, the generation of gene-edited cells with isogenic background, and the scalable production of cellular materials for research [9, 19, 20]. Recently, organoids have aided the investigation of diseases, which can be achieved with a homogeneous and well-defined iPSC-derived EC line [21].

The monolayer differentiation method is most widely used in stem cell research. To understand how this method came into use, one should take a brief look into the achievements of the last 20 years. In 2001, Kaufman et al. succeeded in producing CD34 positive (+) hematopoietic precursor cells from hESCs by co-cultivation with mouse bone marrow stromal cell lines [22]. Later, it was discovered that mesoderm lineage induction by the embryoid body (EB) method led to cardiovascular progenitor cells from which ECs could be produced [23–25]. Then, in 2009, Choi et al. and Taura et al. demonstrated that ECs could be derived from hiPSCs [26, 27]. Until now, different techniques have been reported to improve the efficiency and quality of iPSC-derived ECs, in terms of characteristic markers and function. In the following review, we will highlight and discuss the various differentiation techniques that have been in use for approximately 10 years, including their advantages and disadvantages (Table 1).

**Table 1:** Summary of publications, methods including the growth factors that have been used, disadvantages and advantages and performed analysis

Year	Cells	Format	Growth factors	Analyses	Advantages	Disadvantages
2020 [54]	NCSCs (ECs) VSMCs	grafts	bFGF, TGFb1	NCSCs: P57+, NES+, VIM+,HNK1+, staining: phalloidin + vinculin for cytoskeleton organization in response to substrate stiffness; SMA+ after TGFb treatment; grafts: H&E staining, Verhoeff’s staining, NuMA+, MHC+, Instron mechanical test; CD31+ staining: EC differentiation capacity from NCSCs	VSMCs with enhanced mechanical properties	no lineage specification; heterogeneity
2019 [57]	ECs	monolayer	VEGF, bFGF, shear stress/flow	after shear stress: qPCR: CD309+, CD144+, arterial markers: EPHB2+, NRP1+, staining: CD13+, vWF+, CD144+; tube formation capacity in matrigel	better maturation of ECs; different reactions by arterial/veinal ECs	lack of VSMCs in the model
2019 [9]	ECs PCs	organoid	CHIR99021, BMP-4, VEGF-A, forskolin	staining: CD31+, UEA-I+, PDGFRb+, SMA+; perfusion: FITC-Dextran, MRI of transplants	3D vessel structure, lumen, perfusable	lack of VSMCs in the model; missing GMP standards
2017 [37]	ECs	monolayer	Activin A, bFGF, BMP-4, 8bromo-cAMP, VEGF, EGF	sorting for CD31+, CD144+ and CD309+; stimulation method and stimulation-elimination method; ECs show tube formation capacity in matrigel; ECs are capable in AcLDL uptake	sorting for purity; first description of the stimulation method and stimulation-elimination method; functional ECs	complex protocol
2017 [38]	ECs CMCs	monolayer	CHIR99021, Activin A, VEGF, BMP-4, bFGF	FACS analysis for CD31+, CD34+ and CD144+; hemogenic mesoderm derived ECs have the capacity for erythroid and myeloid lineages based on hematopoiesis assays; EC lumen formation in collagen gel	pure population; directed differentiation due to polarized mesoderm induction; no sorting needed	conditions are not chemically defined, B-27 supplement, and recombinant proteins
2016 [47]	VSMCs	monolayer	VEGF-A, bFGF, PDGF-BB, TGFb1, B-27	contractile: MHC11+, CNN1+, TAGLN+, synthetic: COL-I+, CX43+, VIM+; proliferation & migration increased in synthetic SMCs: contraction as carbachol treatment response: increased in contractile SMCs	phenotype (synthetic & contractile) specific VSMCs	heterogeneity of VSMCs
2015 [36]	ECs VSMCs	monolayer	CP21R7, BMP-4, VEGF, forskolin, PDGF-BB, Acticin A	endodermal progenitors: SOX17+; ECs sorted for: CD144+, CD309+, CD31+, CD34+, CD105+; CD43-/CD45-; VSMCs: PDGFR+, alpha-SMA+, myosin IIb+: transcriptomics reflected vascular cells similar to primary cells; tube formation capacity	checkpoint control by FACS sorting ECs; high purity VSMCs	heterogeneity of ECs; subset of ECs expressed SMA, few mechanical endurance
2014 [41]	VSMCs	monolayer	Activin A, bFGF, SB431542, LY294002, bFGF, BMP-4, PDGF-BB, TGFb1	early mesoderm: brachyury+, NE-lineage: PAX6+, NES+; PM-lineage: TCF1+; LM-lineage: CD309+; SMCs: CNN1+, TAGLN+, MYH11+	checkpoints for mesodermal lineage; directed, lineage specific differentiation of iPSCs into VSMCs	time-consuming
2014 [35]	ECs PCs	monolayer	Activin A, BMP-4, VEGF, CHIR99021, SB43152, bFGF	sorting for CD31+; IF positive for CD144, CD31, vWF and LYVE1; first description of simultaneous derivation of ECs and pericytes; in a zebrafish xenograft model ECs were able to incorporate into developing as well as established vasculature	sorting for purity; functional ECs; validation in in vivo model	generated ECs express less arterial and venous markers than HUVECs; ECs express lymphatic markers
2013 [30]	ECs	EB	bFGF, Activin A, BMP-4, VEGF, apo-transferrin, insulin, EGF, IGF-1, hydrocortisone, retinoic acid	sorting for CD309+; IF positive for CD31, CD144, eNOS, and vWF; ECs are capable in DiI-ac-LDL uptake; ECs show tube formation capacity in matrigel	sorting for purity; functional ECs	low efficiency: many inductors are needed
2013 [32]	ECs	EB	comercial EC growth supplement	sorting for CD144+; IF positive for CD144, CD309, and CD141, eNOS, Ang2, vWF; ECs are capable in AcLDL uptake; ECs show tube formation capacity in matrigel; proinflammatory stimuli induce an activated proinflammatory phenotype	functional ECs; proinflammatory testing	low efficiency; heterogeneity; undirected differentiation
2012 [31]	ECs HPCs	EB	BMP-4, PD98059, SCF, VEGF, Flt3L, bFGF, IL-3, IL-6	hBMMSC-derived iPSCs to produce CD34+ progenitor; sorting for CD34+; IF positive for CD31 and CD144; ECs show tube formation capacity in matrigel	functional ECs	low efficiency; heterogeneity; many inductors are needed
2011 [28]	ECs	EB	VEGF, bFGF	sorting for CD31+; ECs are capable in DiI-ac-LDL uptake, ECs show tube formation capacity in matrigel	functional ECs	low efficiency; heterogeneity
2011 [29]	ECs	EB	BMP-4, VEGF	sorting for CD31+; IF positive for CD309, CD31, CD144, and eNOS; in vivo application of iPSCs in a murine model, ECs are capable in AcLDL uptake	functional ECs; in vivo application of iPSCs in a murine model	low efficiency; heterogeneity
2010 [34]	ECs	monolayer	N2 supplement, B27 supplement, StemPro-34 SFM, VEGF	sorting for CD309+ and CD144+; iPSC-derived ECs have a greater wound healing capacity and oxidative stress tolerance than HAECs; IF positive for eNOS, CD31 and CD144	functional ECs	low efficiency; use of mouse feeder cells; undirected differentiation
2010 [33]	ECs VSMCs	monolayer	PD98059, BMP-4, VEGF-A, bFGF	sorting for CD34+; direct differentiation based on mesoderm induction; ECs show CD31 and CD105 expression, IF positive for CD31, VWF, CD144, ANG2, and CD309, ECs show tube formation capacity in matrigel; ECs are capable in AcLDL uptake	feeder- and serum-free system; direct differentiation; functional ECs	low efficiency
2009 [40]	VSMCs	monolayer	retinoic acid	staining: SMMHC+, alpha-SMA+; sorting SMMHC; qPCR: MYOCD+, TAGLN+; alphaSMA+, SMMHC; carbachol-induced contraction positive for ca. 80% of cells	fast differentiation of iPSCs into VSMCs	undirected and spontaneous differentiation; heterogeneity
2009 [26]	ECs HPs MLCs	co-culture	bFGF, ECGF	sorting for CD34+, CD31+ and CD43-; IF positive for CD144; ECs in FACS analysis positive for vWF, CD309, CD31, CD49d, and CD105; ECs show tube formation capacity in matrigel	co-culture of various vascular cell types	very low efficiency; heterogeneity; contamination due to co-culture
2009 [27]	ECs MCs	co-culture	VEGF	sorting for CD309+ and CD144+; ECs formed a network-like structure in matrigel; IF positive for CD31 and eNOs	co-culture of various vascular cell types	very low efficiency; heterogeneity; contamination due to co-culture

*MCs* mural cells, *HPs* hematopoietic progenitors, *MLCs* mesenchymal lineage cells, *HPC* hematopoietic cells, *PCs* pericytes, *EB* embryoid bodies

### Co-culture Systems

In 2009, Choi et al. and Taura et al. were the first to successfully generate ECs from hiPSCs. They showed very similar properties regarding marker expression, tube formation, and cell behaviour. Both groups used co-culture techniques, in which stem cells (SCs) were cultivated together with mouse bone marrow stromal cell lines, such as OP9 or S17, for 8 to 10 days. Feeder cells provided the SCs with an environment for spontaneous differentiation into CD34+ hematoendothelial precursors cells (HEPs). By using magnet-activated cell sorting (MACS) CD34+ cells were specifically isolated. Subsequently, the sorted cells could be differentiated in the direction of the EC lineage by culturing in EC medium supplemented with basic fibroblast growth factor (bFGF) and vascular endothelial growth factor (VEGF). The groups showed that these cells expressed characteristic EC markers such as CD144, vWF, CD309, CD31, CD49d, and CD105, but the total differentiation efficiency was very low [26, 27]. The co-culture systems used to derive hematopoietic lineages from iPSCs have considerable disadvantages. In addition to their low EC differentiation efficiency, these cultures required the support of animal feeder cells. Due to immunogenicity, this would make implantation into patients practically impossible. Moreover, the co-culture method sometimes required long differentiation times and led to high cell heterogeneity. The latter leads to a mixture of different cell subpopulations, which accordingly, may bias the study results.

### Embryoid Body Systems

The use of EBs to obtain ECs from ESCs has been extensively researched [24, 25]. In 2011, Li et al. and Rufaihah et al. used the EB technique to generate hiPSC-derived ECs [28, 29]. Briefly, iPSC colonies are cultivated in ultra-low binding tubes for several days to form cell aggregates. In this state, internal cells not exposed to the external environment are triggered to undergo spontaneous differentiation. After 4 to 12 days of spontaneous differentiation, the EBs were transferred into adherent conditions supplemented with VEGF and bFGF for another 3 to 10 days to form ECs [28, 29].

Later, this method was refined by culturing the EBs for 4 days with bone morphogenetic protein 4 (BMP-4) and VEGF-A, followed by differentiation into EC subtypes with slight culturing modifications. For a heterogeneous population of ECs, 4-day-old EBs were re-attached and cultured with VEGF-A alone. For arterial EC differentiation, the EBs were cultured with the addition of 8-Bromoadenosine cyclic adenosine monophosphate (8Br-cAMP). For venous EC differentiation, EBs were cultured with VEGF-A alone. For lymphatic EC differentiation, EBs were cultured with BMP-4, VEGF-C, and angiopoietin-1 [30].

In 2012 Xu and colleagues published an EC differentiation method using a chemically defined cocktail [31]. They used stem cell factor (SCF), FMS-like tyrosine kinase 3 ligand (FLT3L), and VEGF during EB formation for mesoderm induction. CD34+ induction was achieved with the addition of bFGF, IL-3, IL-6, VEGF, FLT3L, and SCF. After EB dissociation and sorting, the CD34+ cells were cultured with VEGF-A to initiate differentiation into ECs. This approach was not efficient, yielding 13% CD34+ cells, and not cost-efficient due to the number of factors required [31].

To generate a pure culture of ECs with the same functional phenotypic plasticity of mature primary vascular endothelium, Adams et al. dissociated the EBs, sorted, and subsequently cultivated the CD144+ cells in EC medium [32]. The sorting step was necessary because CD31+ and CD309+ cells do not express CD144. This result is problematic for previous studies because CD144 is a highly specific marker of EC identity. It is important to note that the EB differentiation efficiency from CD144+/CD31+ cells was 18% [32].

Just like co-culture systems, the EB method has many disadvantages. The EB method yields to a highly heterogeneous cell population with low EC efficiency. Based on these reasons, the EB method is unfavourable for high-throughput models, which need a large number of homogenous cells.

### Monolayer Systems

Park et al. 2010 were among the first to develop a monolayer system that did not require additional feeder cells. Instead, the authors demonstrated that mesoderm induction from PSCs is a key step towards generating hematoendothelial precursor cells [33]. Commitment into the mesoderm lineage did not require the formation of EBs, or co-culture with mouse bone marrow stromal cell lines; instead, it could be induced by BMP-4 alone. Additionally, PD98059 (a potent and selective inhibitor of MAPkinase kinases) was shown to increase mesoderm induction. Furthermore, they found that BMP-4 did not prevent further differentiation of CD34+ cells. However, the overall differentiation efficiency of CD34+ cells was only 13%. For differentiation of ECs, treatment with bFGF and VEGF-A was sufficient [33].

In the same year, Homma and colleagues published an undirect differentiation method in which iPSCs were incubated with N2 and B27 supplement for three days [34]. After another three to five days with StemPro-34 SFM and VEGF, the CD309+ and CD144+ cells were sorted and used for functional analysis. Experimentally, the authors were able to show that iPSC-derived ECs have a greater wound healing capacity and oxidative stress tolerance than human aortic endothelial cells (HAECs). Moreover such ECs have a comparable localization of eNOS, CD31 and CD144 in immunofluorescence analysis compared with HAECs [34].

Orlova and colleagues 2014 showed that CHIR99021 (an inhibitor of the enzyme GSK-3) was sufficient to start mesoderm induction in monolayer culture, while Patsch et al. 2015 reported that the addition of BMP-4 increased the yield [35, 36]. Further, Patsch et al. 2015 presented an optimised differentiation method for generating ECs. First, the researchers treated hiPSCs with BMP-4 and CHIR99021 up to day 4 to generate mesodermal cells, followed by a cocktail consisting of Forskolin and VEGF-A to induce ECs up to day 6. For the maturation of the ECs, the researchers continued treatment with VEGF-A. This protocol circumvented the use of feeder cells or EBs, and was shorter, more cost-effective, and yielded 30 times more ECs from iPSC-derived mesodermal SC precursors. From 1 million iPSCs, 30 million ECs can be produced. An additional advantage of this protocol is that the EC cocktail can be modified to an SMC cocktail after mesoderm induction to produce SMCs instead of ECs. This modification consists in the use of N2B27 medium plus PDGF-BB and Activin A from day 4 for SMC induction and from day 6 additionally heparin for maturation into VSMCs [35, 36].

Two years later, Ikuno et al. published an enhanced differentiation protocol that could yield 10 times more ECs than the Patsch method [37]. This was achieved by the addition of cAMP as an inducer for early mesoderm and early EC commitment. This was based on the activation of protein kinase A (PKA) by cAMP, which resulted in increased expression of VEGFR2 and PKA-activated CREB-mediated activation of Etv2/ER71 (an ETS transcription factor) for early EC commitment. Another interesting aspect of the Ikuno et al. study is the so-called stimulation-elimination method. After mesoderm induction, a stimulation step is performed for 2 days by treatment with VEGF and cAMP, and then all non-responder cells are eliminated by VEGFR2+ Fluorescence-activated cell sorting (FACS). The responder cells were then stimulated for another 3 days. By eliminating the non-responder cells early, the resulting population was 99% pure ECs. This stimulation-elimination method eliminates the need to purify the cells at a mature cell stage, which in turn increases the yield and viability of ECs [37].

**Fig. 3:**
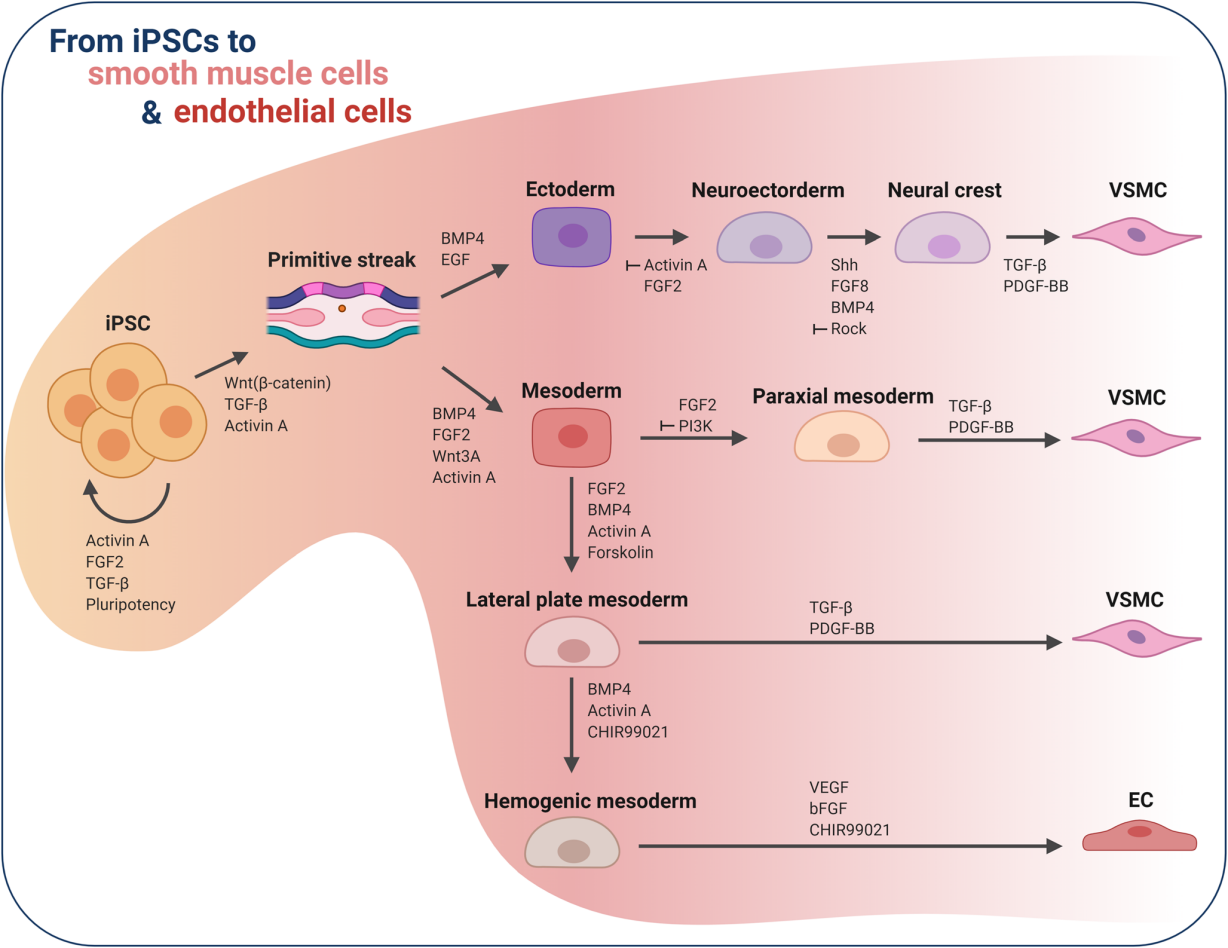
Embryological differentiation pathways of VSMCs and ECs

In the same year, Palpant and colleagues demonstrated the importance of cellular polarization for differentiation of mesoderm subtypes [38]. They used different concentrations of Activin A and BMP-4 to generate cardiogenic and hemogenic mesoderm, respectively. From these subtypes, both cardiomyocytes and functionally diverse ECs could be differentiated (Fig. 3). This work clearly showed the importance of considering the endothelial lineages. Depending on the desired function or target site, the different lineages must be considered. Therefore, it is necessary to know in advance which endothelial subtype, whether endocardial, vascular (venous or arterial), or lymphatic, is required [30, 38, 39].

The large amount of publications and differentiation protocols reflects the interest in this developing field, with a new focus on 3D cultures of organoids and tissues. The optimised monolayer-based methods of EC generation will continue to be used in the field, based on reliability and high-yield differentiation, which could also be applied at an industrial scale. Nevertheless, further studies will be necessary to elucidate lineage specificity, so that it is possible to differentiate functionally distinct ECs that are tailored to their destination. Regarding therapeutic application, further knowledge is needed to reduce the use of immunogenic substances, such as fetal bovine serum (FBS), or animal-derived coating matrices.

## iPSC-Derived Vascular Smooth Muscle Cells

Obtaining primary vascular SMCs from patients is difficult and highly invasive. A better alternative is to differentiate vascular SMCs (VSMCs) from patient-derived iPSCs. However, to date, only a few VSMC differentiation protocols have been reported (Table 1). In 2009, Xie and colleagues published a method using all-trans retinoic acid (RA) for the differentiation of murine SCs into VSMCs [40]. This method is also effective for human PSCs (Trillhaase, 2020). Importantly, treatment of iPSCs with RA for 10 days leads to spontaneous differentiation into VSMCs. Therefore, the resulting VSMC population will be a heterogeneous mix of SMCs from different embryonic origins. Nonetheless, these VSMCs express SMC-specific markers like alpha smooth muscle actin (SMA) and smooth muscle myosin heavy chain (MHC) [40].

During embryonic development, VSMCs originate from the neuroectoderm (NE), lateral-plate mesoderm (LM), and paraxial mesoderm (PM) lineages [41]. In 2014, Cheung and colleagues published a protocol that facilitated lineage specification for VSMC differentiation [41]. To specify the NE, human PSCs were treated with FGF2 and SB431542 for 7 days [41]. For LM and PM specification, the PSCs were first differentiated into early mesoderm by supplementation with FGF2, LY294002, and BMP-4 for 1.5 days, followed by either FGF2 and high LY294002 supplementation, or FGF2 and high BMP-4 supplementation, for PM and LM specification, respectively [41]. To differentiate SMCs, the specified cells were then treated with platelet-derived growth factor (PDGF-BB) and transforming growth factor beta (TGFbeta1) [41]. This method closely mimicked embryonic development as reported by Sinha and colleagues [42] and can be used to investigate VSMCs from a specific lineage (Fig. 3).

The vascular system is made up of VSMCs derived from different lineages. Cells of the NE lineage give rise to VSMCs in the ascending aorta, the aortic arch, and the pulmonary trunk [43]. VSMCs from the PM are found in the descending aorta [42]. VSMCs from the LM are first located in the pro-epicardium in the looped-heart stage of embryogenesis and then settle at the venous pole [42].

**Fig. 4:**
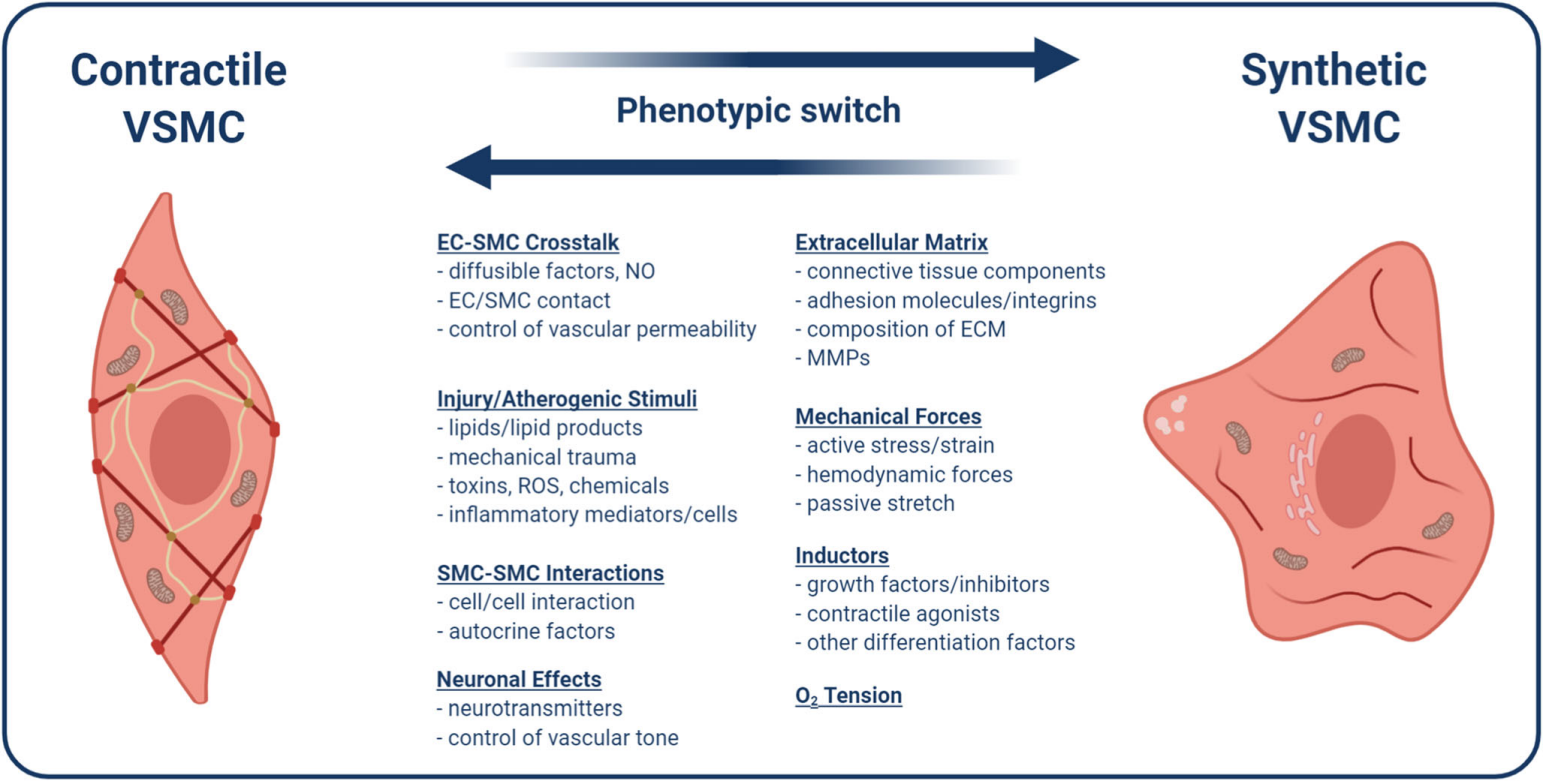
VSMC phenotypic switch between contractile and synthetic phenotype. Various factors contribute to VSMC phenotypic switch, including cell interactions between ECs and SMCs, mechanical forces, ECM composition, oxygen (O_2_), neuronal effects, injury, and inflammation

In the vascular system, VSMCs are an important regulator of blood flow and pressure [43]. Due to their contractile ability, they are responsible for vaso-dilatation and constriction, and blood flow dispersion [44, 45]. An important characteristic of VSMCs is their ability to switch from a contractile to a synthetic phenotype. The synthetic phenotype is mediated by PDGF, bFGF, insulin-like growth factors, epidermal growth factor, α-thrombin, factor Xa, angiotensin II, endothelin-1, and unsaturated lypophosphatidic acid [45]. On the other hand, the contractile phenotype is promoted by the expression of heparin, TGFβ, and angiotensin II and IGF1 [45, 46]. Generally, VSMC phenotypic plasticity and the switch from contractile to synthetic phenotype are characterized by reduced expression of VSMC-selective differentiation markers, such as Calponin (CNN1), Transgelin (TAGLN), Caldesmon (CALD1), SMA, or smoothelin. Further they show increased proliferation rate, increased migration, and the synthesis of extracellular matrix (ECM) components required for vascular repair (Fig. 4) [44].

In 2016, Yang et al. published a method that allows the differentiation of phenotype-specific VSMCs [47]. First, iPSCs are split 2 days prior to differentiation [47]. For differentiation into the synthetic phenotype, progenitors are treated with VEGF-A and FGF2 for 7 days in the absence (d3–7) or presence (d7–10) of B27 supplement [47]. From day 10, these cells were then treated with PDGF-beta and TGF-beta for 4 days to specify synthetic VSMC properties. Purification of iPSC-derived synthetic VSMCs was performed using 4 mM lactate. For the differentiation into VSMCs with the contractile phenotype, cells were treated with VEGF-A and FGF2 in the absence of B27 for 4 days. Starting as early as from day 7, PDGF-beta and TGF-beta was applied to induce VSMCs into the contractile phenotype. Purification was again performed on day 14 with 4 mM lactate [47]. Purification of cells increased the amount of SMA+ cells from approximately 40% to 95% [47]. Further, the VSMC markers MHC11, CNN1, and TAGLN, were expressed significantly more in contractile compared with synthetic iPSC-derived VSMCs [47].

The contractile phenotype of VSMCs is predominantly found in the tunica media, which is required for the regulation of vascular tone [48]. Contractile VSMCs are characterized by a functional contractile apparatus, low proliferation rate, low connective tissue, and a response mechanism to neurotransmitters such as acetylcholine and norepinephrine [42, 45]. The synthetic phenotype, however, is characterized by fibroblast-like morphology, excretion of high amounts of ECM, high proliferation rate, and decreased expression of contractile proteins [42, 45, 49].

It remains unclear as to whether VSMC phenotypic plasticity contributes to the onset and progression of vascular diseases, such as atherosclerosis [42, 44, 48]. However, it is known that vascular calcification or mineralization contribute to atherosclerosis, and takes place within the plaque and the fibrous cap, as well as in the media [50].

VSMCs are often involved, if not the leading force, in this process [51–53]. As primary VSMCs quickly lose their contractile properties in vitro [43], it is necessary to be able to differentiate VSMCs in an origin-specific manner from PSCs. Any in vitro findings therefore need to be verified in an in vivo model [44]. Further, primary VSMCs lose their proliferative potential with increasing donor age, therefore they are not always a reliable source of cells [43]. Taking all this into consideration, PSC-derived VSMCs are the best option to generate high cell numbers in vitro*,* eliminate patient risk, and reduce loss of contractility [43, 44]. Further, the in vitro differentiation of PSCs into VSMCs from different origins are important to examine their contribution to disease progression and outcome. 3D organoid models represent the next advancement in the field, as they resemble the structural and functional complexity of the vascular system including cell–cell interactions.

## Vascular Organoids

As described earlier, various 3D models of organs and tissues have been developed within the last 10 to 15 years. This includes the brain, lung, liver, and intestines. These 3D cell culture models not only mimic embryonic development, but also better resemble the physiological situation than 2D systems. Three protocols were developed within the last 5 years to differentiate vascular cells from iPSCs and pave the path for their use in 3D grafts [54]. All three protocols successfully lead to the generation of vascular ECs or VSMCs via different pathways. However, all of the three protocols display weaknesses, such as missing capability in mechanical durability, missing VSMCs, cell heterogeneity, and immaturity of cells [54]. Therefore, they were inadequate for use in 3D grafts [54]. Another approach towards 3D engineered vascular tissue was made by constructing tissue-engineered vascular grafts (TEVGs) which are basically artificial blood vessel constructs embedded in matrix. Different cell types, such as stromal cells, various ECs, and VSMCs were co-cultured in a hydrogel and formed vessel-like structures that are perfusable [54]. Even though the TEVGs showed some more levels of physiological properties than the before-mentioned protocols, they were still not applicable in clinics as they showed low plasticity and few connections to the host blood system [54].

Additionally, two models were developed potentially suggesting clinical application of human iPSC-derived vascular cells. In 2011, Lin and colleagues developed a model for human vascular networks against anemia. Endothelial colony-forming cells (ESFCs) and mesenchymal stem cells (MSCs) from anemia patients were co-cultured in collagen/fibrinogen hydrogels and injected subcutaneously in mice. The grafts successfully produced networks of perfused blood vessels expressing human CD31. However, the aim was to investigate whether genetically engineered grafts could cure anemia. Therefore, human erythropoietin was overexpressed in the grafts and implanted in a mouse anemia model [55]. The gene edited grafts successfully formed vessel networks and expressed and secreted erythropoietin into the bloodstream of mice [55]. Further, using a tetracyclin system it was possible to induce gene expression of erythropoietin in the grafts with oral supplementation of doxycyclin in mice. Mice with access to doxycyclin recovered faster after radiation than control mice, showing that implants maintained the ability to produce erythropoietin even after high radiation doses [55]. Further, a mouse model for renal failure was treated with the engineered grafts showing a fast recovery after radiation and doxycyclin treatment even with insufficient renal function. Most importantly, they reported a confined cellular engraftment, as human ECFCs or MSCs could not be detected elsewhere in the mice. In summary, Lin and colleagues managed to achieve a correction of anemia in a mouse model using human endothelial-based vascular grafts with erythropoietin overexpression after only 1 week. This method combines the advantages of ex vivo gene editing and specific cellular engraftments. The authors suggest that their model might lead to the development of an alternative treatment of chronic anemia patients without the need of repetitive, regular injections and therefore, also cost reduction in medical treatment of anemia [55].

Only very recently, Neumeyer and colleagues published another 3D model made of human iPSC-derived ECs of hemophilia A patients that can be transplanted into the subcutaneous site [56]. Hemophilia A is a bleeding disorder caused by dysfunctional coagulation-factor 8 (F8). As F8 is a comparably large gene it is hardly packed into common vectors for gene therapy. Therefore, Neumeyer and colleagues produced iPSC-derived ECs from hemophilia A patients and delivered the functional F8 gene into these ECs. Subsequently, xenografts of the genetically engineered ECs were injected into the subcutaneous space of hemophilic mice. The hemophilic grafts carrying the functional F8 gene formed perfused vessel networks and their lumens were filled with mouse erythrocytes, showing connection to the host blood system [56]. The F8 overexpressing ECs also rescued the clotting deficiency in hemophilic mice, and increased activity of F8 was detected pointing towards efficient release of F8 from the grafts *in vivo* [56]. However, the model also has its weaknesses. The subcutaneous application of human ECs in mice is only stable for 1 week. Longer duration of the experimental setting lead to replacement of human ECs by mouse ECs. Therefore, long-term studies testing EC engraftment in other tissues need to be performed to determine the most suitable site of application to guarantee efficacy over long periods of time for clinical applications. Nevertheless, Neumeyer and colleagues were able to rebuild a hemostasis comparable to healthy animals in only 1 week. Both aforementioned protocols show successful 3D models of vascular cell implants that were able to, at least in an animal model, restore two diseases of the hemostatic system and perspectively offer the basis for the development of gene therapy and even personalized therapy by vascular graft delivery.

Recently, Wimmer and colleagues published the first PSC-derived vascular organoid model [9]. Briefly, human PSCs were differentiated in vitro into mesodermal cells, and then VEGF-A and forskolin were applied for 2 days to induce the vascular lineage [9]. To create a 3D model, Wimmer and colleagues used Matrigel/Collagen hydrogels to stimulate vessel sprouting. The resulting vascular networks were then isolated from the hydrogel and cultured in round-bottom, low binding plates to promote 3D vessel organoid formation [9]. The vascular organoids consisted of ECs and Pericytes (PCs). This protocol allowed the culture of 3D vascular organoids in 96 well plates over a long period, and showed significant lumen formation [54]. After transplantation into the mouse kidney, the organoids connected with the host vessels, allowing perfusion. This provided evidence that the PSC-derived vascular organoids were functional [9]. However, some important measures are missing that are required for good manufacturing practices in order to use the organoids in clinical approaches. Cellular heterogeneity was not assessed and might combat the reproducibility of the method. Further, a control checkpoint is missing that would be required for clinical application [54].

The application possibilities of the organoids are numerous, starting from investigating high blood pressure and shear stress effects on ECs, to the effects of inflammatory factors on SMC behaviour and phenotype switching, or cross-talk between ECs and VSMCs during stress. Furthermore, transplantation of pre-vascularized tissues could accelerate wound healing by promoting the connection of the transplant with the surrounding host tissue [58]. In future, it would be advantageous to adapt the vascular organoids to contain VSMCs beneath the already established cell types of the vascular system ECs and PCs to better resemble the physiological environment.

Recently, other PSC-derived organoid models have been used to investigate disease onset, progression, and pathology. For example, human mini-brains have been used to analyse the genetics of microcephaly, including its connection to the Zika-virus [59]. Very recently, researchers used PSC-derived organoids to investigate the infection strategy of the SARS-CoV2 virus for identification of potential drug candidates [59]. Human organoids models have already been used to investigate infectious diseases, cystic fibrosis, and cancer [59], and the list of applications is still growing.

In the field of cardiovascular research, 3D cell culture systems, such as human organoids, closely mimic the complex in vivo physiology of the vascular system, allowing the investigation of genetics, function, and disease pathophysiology. Use of these model systems is an ideal alternative to reduce the use of experimental animals and their derivatives and allows us to examine human pathophysiology.

However, the field of human iPS-derived organoids is still very young. Various difficulties occurring along the way must be addressed in order to make this method reliable and clinically applicable. First, the cell heterogeneity within iPSC-lines and even clones have to be tackled, and heterogeneities within and between the organoids have to be addressed. Further, the lack of perfusion in vitro is a major obstacle, as perfusion of vessels is needed to get rid of waste, to evenly distribute nutrients, and most important to force EC maturation by shear stress [54]. Additionally, blood components as well as immunological cell types should be considered to be integrated into the organoids to increase translatability and to resemble a more physiological situation. Finally, we propose that the biggest challenge that needs to be addressed first in the organoid research is to replace animal contents. Most protocols rely at least on some kind of animal tissue or components, such as Matrigel or rat collagen [9], decellularized porcine intestine scaffolds [54], or FBS which is still quite common in cell culture labs around the world.

Nevertheless, we believe that iPS-derived vascular organoids hold a great promise in vascular research allowing studies on functional and genetic backgrounds of different disease patterns of coronary artery disease. This technique allows investigations impossible with primary tissue and animal models such as developmental studies on vasculature or high content drug screening on human tissue with an almost unlimited availability.

## Data Availability

Not applicable.
